# Effect of nitrogen fertilization and shading on morphogenesis, structure and leaf anatomy of *Megathyrsus maximus* genotypes

**DOI:** 10.3389/fpls.2024.1411952

**Published:** 2024-07-22

**Authors:** Aline da Rosa Lopes, Nauara Moura Lage Filho, Aníbal Coutinho do Rêgo, Felipe Nogueira Domingues, Thiago Carvalho da Silva, Cristian Faturi, Naiara Caixeta da Silva, Wilton Ladeira da Silva

**Affiliations:** ^1^ Institute of Veterinary Medicine, Federal University of Pará, Castanhal, Pará, Brazil; ^2^ Department of Animal Science, Federal University of Ceará, Fortaleza, Ceará, Brazil; ^3^ Institute of Agrarian Science, Federal University of Jequitinhonha and Mucuri Valleys, Unaí, Minas Gerais, Brazil; ^4^ Institute of Health and Animal Production, Federal Rural University of Amazon, Belém, Pará, Brazil; ^5^ Department of Animal Science, Federal University of Goiás, Goiania, Brazil

**Keywords:** leaf anatomy, morphogenetics characteristics, *Panicum maximum*, silvopastoral system, tiller

## Abstract

The use of exotic grasses of African origin for pastures in Brazil has been a major advancement in livestock production, but little is known about the responses of these grasses to nitrogen fertilizers associated with shading. In this study, the morphogenetic, structural, and leaf anatomical characteristics of Megathyrsus maximus cultivars’ Tamani and Quênia were investigated as a function of N dose and shade. Morphogenetic and structural characteristics and leaf anatomy were studied under three shading levels (0, 30, and 50 %) and four N doses (0, 100, 200, and 300 kg N ha^-1^) to simulate growth in a silvopastoral system. When comparing the cultivars, Quênia was more efficient in terms of phyllochron up to fertilization with 100 kg N ha^-1^. The leaf senescence rate of Tamani was higher than that of Quênia at the 30 and 50 % shade levels. The total area (TA) occupied by leaf tissues decreased in Quênia as a function of the increase in N fertilization, whereas the TA of Tamani did not change. The thickness of the adaxial epidermis was greater in Quênia (0.68 µm) than in Tamani (0.50 µm) when not fertilized. The area occupied by the mesophyll was greater in both cultivars when they received fertilization equivalent to 300 kg N ha^-1^. Quênia grass has a smaller phyllochron than Tamani grass, due to the rapid reconstruction of its photosynthetic apparatus, especially when it receives higher levels of nitrogen fertilization. However, Tamani grass has a greater distribution of plant tissues. The mesophyll area is larger in Tamani grass due to the greater presence of chloroplasts, which facilitates digestion by animals. The Tamani modified the leaf anatomical tissues more significantly in relation to shading, whereas the Quênia modified them in relation to N fertilization, which reinforces the suggestion of a more appropriate use of Tamani in silvopastoral systems.

## 1 Introduction

In Brazil, the use of exotic grasses of African origin in the composition of pastures has greatly increased, making it the second largest beef producer in the world (ABIEC, 2023). However, native grasses that are better adapted to Brazilian edaphoclimatic conditions have increasingly lost space for these exotic species. In the Savanna biome, which occupies approximately 25% of Brazil’s territory, exotic grasses, which are considered more competitive and aggressive, are the most commonly used in pasture areas. Consequently, native species have been reduced or eliminated as a result of competition for natural resources, pushing the ecosystem into a critical conservation state (Musso et al., 2019).

The savannah biome in Brazil is the second largest in terms of land area, behind only the Amazon biome, and is characterized by a predominance of acidic and dystrophic soils with high levels of aluminum and iron and low phosphorus availability. In recent decades, there has been an expansion of livestock in this biome, which has increased the use of exotic grass species of African origin as they are well adapted to this environment, and the environmental conditions of South American savannas are like their original habitat (Musso et al., 2021).

Cattle ranchers prefer pastures composed of grasses of African origin likely due to the adaptation to environmental conditions but also the rapid response of these grasses to fertilization (Oliveira et al., 2020; Santos et al., 2020), and higher forage production (Silva et al., 2017, 2019; Musso et al., 2021), as they are more resistant to cattle trampling and grazing (Klink, 1994) and have a medium to high tolerance to shading (Paciullo et al., 2016; Santiago-Hernandez et al., 2016; Pereira et al., 2021).

Much remains to be learned about the adaptation of these exotic grasses in savanna, particularly when they are exposed to different levels of nutrient availability, for example nitrogen (N). This knowledge is important for optimizing their use, with higher forage production in smaller areas reducing the pressure on native vegetation. In this context, the silvopastoral system (SPS), which increasingly uses exotic grasses of the genus *Megathyrsus* (Marcillo et al., 2021; Pereira et al., 2021), moves ahead of native vegetation. SPS is characterized by the association of trees or arboreal plants with animals in pastures. SPS aims to establish different plant hierarchies, where trees or shrubs are considered key to the stability of the system, providing benefits in terms of soil quality, forage quality, greenhouse gas balance, and animal welfare (Pereira et al., 2021).

One problem with using grasses of the genus *Megathyrsus* in SPS in Brazil is that very little is known about the effects of shade on these plants, especially in association with the use of nitrogen fertilizers. Some studies have shown that shading rates greater than 50% of the incident radiation can affect the biomass production of tropical grasses (Abraham et al., 2014) due to reduced photosynthetic rates (Santiago-Hernandez et al., 2016), in addition to changes in their anatomy and morphology. However, under moderately shaded conditions, plant production can be similar to or, in some cases, higher than that under monoculture or full sun pasture conditions (Paciullo et al., 2014).

Nitrogen fertilization is another widely used pasture management strategy in the Brazilian Savanna because of the severely limited growth of exotic tropical grasses (Silveira et al., 2015). Research has shown that there are benefits to moderately shaded pastures growing in low natural N availability, owing to higher soil moisture in shade compared to soils with no shade. This increased moisture can promote microbial activity in the litter, which accelerates the mineralization of organic matter, thus improving soil N availability. However, for most grasses that require high soil fertilization, such as those belonging to the genus *Megathyrsus*, the N content in shaded environments may be insufficient to meet this requirement (Bernardino et al., 2011).

Environmental and nutritional stress in plants provides responses from the subcellular to the structural and productive levels; therefore, several morphogenetic, structural, and anatomical parameters were evaluated in the two cultivars of *Megathyrsus maximus*, (Tamani and Quênia). The simultaneous study of these responses may help reveal the underlying mechanisms of their morphological responses. Thus, we intended to answer the following questions: (1) Are the morphoanatomical and morphogenetic responses of cultivars different between shade and fertilization treatments? (2) If so, what conditions favor the cultivars? and (3) which cultivar is the most adapted, aiming at a scenario of use in integrated systems with shade and nitrogen fertilization, such as SPS? Since grasses are exotic and very responsive to nutrient supply, especially N, and as there is a lack of information on the relationship between nitrogen and shading in these plants, we hypothesized that the shading of cultivars in a simulated SPS environment would affect the responses derived from N application during the regrowth period, and that this would have implications for the morphogenetic and structural patterns and leaf anatomy of the grasses studied.

## 2 Materials and methods

### 2.1 Cultivars and experimental area

Two cultivars (cv) of *Megathyrsus maximus* (Tamani and Quênia) were used. Their seeds were sown in trays with substrates in October 2018 and placed in a greenhouse with controlled irrigation in order to facilitate germination. Thirty days after sowing, the seedlings were transplanted to the experimental field, and evaluated from January to April 2019. An experimental area at the Federal University of Goiás, Brazil (16°35’ S, 49°16’ W) was used for this purpose. The soil in the experimental area was a Clayey Oxisol and had the following chemical characteristics in the surface layer from 0 to 20 cm: pH (CaCl_2_) = 5.1; P (Melich I method) = 2.0 cmolc dm^-3^; K = 0.092 cmolc dm^-3^; Ca = 3.1 cmolc dm^-3^; Mg = 1.1 cmolc dm^-3^; Al = 0.0 cmolc dm^-3^; H+Al = 2.9 cmolc dm^-3^; cation exchange capacity = 7.19 cmolc dm^-3^; and saturation base = 60%. The local climate is classified as Aw (Alvares et al., 2013), which is typical of a tropical savannah climate with dry winters, and 1412 mm average annual rainfall in the last 10 years. From October 2018 to April 2019 the total rainfall was 1062 mm, and average air temperatures ranged from 25.0 to 26.5°C.

The seedlings were transplanted to the experimental units in the field, each with an area of 1 m^2^ (1.0 x 1.0 m). There were 20 plants per experimental unit, with row and interplant spacing of 25 cm.

### 2.2 Experimental design and management

A completely randomized design was used in a 2 × 3 × 4 factorial scheme with three replicates. The two grass cultivars (*Megathyrsus maximus* cv. *Tamani, and Megathyrsus maximus* cv. Quênia) were subjected to three levels of artificial shading (SH) (0, 30, and 50%) and four nitrogen fertilization doses (NF) equivalent to 0, 100, 200, and 300 kg ha-1 year-1, resulting in a total of 72 experimental units.

Shading was achieved using a polypropylene shading cloth of different mesh sizes, which was fixed at a height of 1.5 m above the soil surface and on the sides of the experimental units. Polypropylene clothes were used to simulate the shading observed in the SPS.

Nitrogen doses, except for the zero dose, were applied three times in all treatments using urea (45% N) during the experimental period. Fertilizer was applied in December 2018, and January and February 2019.

The plots in the field were cut whenever they reached an average height of 55 cm for the Tamani cultivar and 70 cm for the Quênia cultivar, during the 120-day harvest period from January to April. Cutting was stopped at stubble heights of 25 cm for Tamani and 35 cm for Quênia. These cutting heights were used to simulate cattle grazing under rotational stocking (Tesk et al., 2020; Pereira et al., 2021). To measure the pastures height, a centimeter-graduated ruler was used at five points per plot. The different cutting heights associated with the treatments resulted in different harvest numbers. Quênia cultivars were harvested four times at fertilizations of 0 and 100 kg N ha^-1^ and 0 and 30% shading, and five times at 200 and 300 kg N ha^-1^ and 50% shading. The Tamani cultivar was harvested three times at fertilizations of 0 and 100 kg N ha^-1^ and 0% shading, and four times at 200 and 300 kg N ha^-1^ and 30 and 50% shading.

### 2.3 Measurements

#### 2.3.1 Morphogenetics and structural characteristics

To evaluate the morphogenetic and structural characteristics of both the cultivars, two tillers from different plants were selected, identified in each experimental unit, and monitored weekly throughout the experimental period. The lengths of the expanding, expanded, and senescent leaf blades, and the length of the pseudostem (sheath and stem) were measured during the evaluations. Leaves were classified as expanded if the ligule was completely visible or as expanding if the ligule was not yet visible. Leaves were classified as senescent if any part of the leaf blade was yellow. Leaves in which more than 50% of the blade length was affected by senescence were considered dead.

Leaf blade length was measured according to the leaf development stage. For expanded leaves, the distance from the tip of the leaf to the ligule was considered. For expanding leaves, the procedure was similar except that the reference point was the ligule of the youngest, fully expanded leaf (Duru and Ducrocq, 2000). For senescent leaves, the effect of senescence on blade length was considered. The pseudostem length was measured as the distance between the soil surface and the ligule of the youngest fully expanded leaf.

After each grass cut, two new tillers were selected per experimental unit to continuously monitor the morphogenetic and structural characteristics. Based on these assessments, the following morphogenetic and structural variables were calculated.

The leaf appearance rate (LAR, leaf tiller^-1^ day^-1^) was calculated by dividing the average number of leaves per tiller by the number of days in the evaluation period.

Phyllochron (PHY, days leaf^-1^) was calculated as the inverse of LAR.

The number of live leaves per tiller (NLL) was determined by adding the number of expanding, expanded, and senescing leaves per tiller.

Leaf lifespan (LLS, dias) was calculated using the duration between the emergence of the leaf and its complete senescence, according to the equation LLS = NLL x PHY (Lemaire and Chapman, 1996).

The leaf elongation rate (LER, cm tiller^-1^ day^-1^) was calculated by dividing the length of the expanding leaf blades per tiller by the number of days in the evaluation period.

The leaf senescence rate (LSR, cm tiller^-1^ day^-1^) was calculated by dividing the length of the leaf blade during senescence by the number of days in the evaluation period.

Final leaf size (FLS, cm) was defined as the length of the expanded leaf blades.

The stem elongation rate (SER, cm tiller^-1^ day^-1^) was calculated by dividing the difference between the final and initial stem lengths by the number of days during the evaluation period.

At the start of the experimental period, a tussock was chosen from each experimental unit and all tillers were counted and identified using wires of a predetermined color. When the grass reached the predetermined cutting height, the already-marked tillers were counted, and the new tillers were counted and marked with wires of different colors. Each new marking with a differently colored wire represented a new generation. This method allowed for the estimation of the tiller population in all generations, enabling the calculation of the respective natality and mortality rates (Equations 1 and 2).


(1) $$TER=\frac{\frac{NNTcg}{NLTpg}}{RP}\;\;x\;100$$

(2) $$\text{TMR}=\frac{\frac{\text{NDT}}{\text{NLTpg}}}{\text{EP}}\times 100$$

Where:

TER = tiller emergency rate, in tillers 100 tillers^-1^ day^-1^

TMR = tiller mortality rate, in tillers 100 tillers^-1^ day^-1^

NNTcg = number of new tillers marked in the current generation

NLTpg = number of live tillers marked in the previous generation

NDT = number of dead tillers

EP = regrowth period

#### 2.3.2 Leaf anatomy

When the grass reached cutting heights of 55 cm and 70 cm (Tamani and BRS Quênia, respectively), three leaf blades were collected from random vegetative tillers for sampling. These samples were taken only in the treatments without fertilization, 300 kg N ha^-1^, without shading and with 50% shading. The last expanded leaf blade was selected and cut into the collar region. Once collected, the leaf blades were divided along their midsections, yielding fragments measuring approximately 1 cm. These were then placed in 1.5 mL Eppendorf tubes and covered with AFA solution (a combination of alcohol (90%), formaldehyde (5%), and acetic acid (5%)) before being set aside for histological preparation.

Leaf blade fragments were gradually dehydrated using an alcohol series to preserve the cell structures. After dehydration, paraffin was used to embed the material, which was then cross-sectioned at 7 μm using a rotary microtome and fixed to slides for staining. The cell walls were stained with Toluidine Blue and Basic Fuchsin (Kiernan, 2008) to aid visualization using software. The proportions of various leaf tissues were determined using a binocular optical microscope in conjunction with the Axion Vision Image Analysis Software, version 3.1. The total cross-sectional area of the blade projected onto the video was subsequently measured using software Axion Vision, version 3.1.

Measurements were taken in micrometers (µm) to determine the thickness of the epidermis and the areas that the other tissues occupied (µm^2^). Tissues were classified as follows: adaxial and abaxial epidermis (ADE and ABE), sclerenchyma (SCL), vascular bundle parenchyma sheath (VBPS), cell wall parenchyma sheath (CWPS), vascular tissues (VT) and intercellular spaces (IS). The mesophyll (MES) was calculated as the difference between the total cross-sectional area (TCSA) and the area of all other tissues.

### 2.4 Statistical analysis

The data were analyzed using the means of the grass regrowth periods. Analysis of variance was performed using the PROC MIXED procedure in the SAS statistical package (SAS, 2001). Cultivar (CV), shading (SH), and nitrogen fertilization (NF) were considered fixed effects, whereas blocks and experimental errors were treated as random effects. When there was a significant main effect (p ≤ 0.05) of NF on the morphogenetic and structural variables, orthogonal polynomial contrasts were used to evaluate the linear (L), quadratic (Q), and cubic (C) effects. Linear and quadratic effects were used for the SH. The means for cultivars and anatomical variables were compared using the F-test, which was considered significant at p ≤ 0.05.

## 3 Results

### 3.1 Morphogenetics and structural characteristics

PHY, TER, and TMR were affected (p ≤ 0.05) by the interaction with CV x NF (**Table 1**). The Quênia cultivar exhibited greater efficiency than Tamani, with the lowest PHY values observed at up to 100 kg N ha^-1^. PHY decreased linearly from 13.00 to 9.07 days leaf^-1^ with increasing nitrogen doses, indicating that Tamani requires more N in the soil to reduce the leaf emergence interval.

**Table 1:** Effects of the interaction between cultivar and nitrogen fertilization on morphogenetics and structural characteristics of tropical grasses of the genus *Megathyrsus*.

Cultivar	Nitrogen fertilization (kg N ha^-1^)				Contrast	Mean	SEM
	0	100	200	300			
PHY (days leaf^-1^)							
Quênia	7.93 b	7.83 b	9.02 a	8.92 a	ns	8.44	
Tamani	13.00 a	9.76 a	9.80 a	9.07 a	L	10.61	
Mean	10.46	8.73	9.91	8.99			
SEM							0.52
TER (tillers 100 tyllers^-1^ day^-1^)							
Quênia	0.35 a	0.36 b	0.51 b	0.82 b	Q	0.51	
Tamani	0.29 a	0.54 a	0.74 a	1.08 a	L	0.66	
Mean	0.32	0.44	0.62	0.95			
SEM							0.04
TMR (tillers 100 tyllers^-1^ day^-1^)							
Quênia	1.01 a	1.08 a	1.24 a	1.58 a	Q	1.23	
Tamani	1.08 a	1.03 a	1.11 b	1.18 b	Q	1.10	
Mean	1.05	1.05	1.17	1.38			
SEM							0.03

PHY, Phyllochron; TER, tiller emergency rate; TMR, tiller mortality rate; L, linear effect; Q, quadratic effect; ns, not significant (P>0.05); SEM, standard error of the mean. Means followed by the same letter within columns are not different (P>0.05).

The tiller emergence rates for Tamani were higher (p ≤ 0.05) than those of Quênia when the doses of N were applied, with Quênia showing a quadratic response and Tamani showing an increasing linear response with increasing NF applied. The TMR of Quênia cultivar was higher (p ≤ 0.05) compared with Tamani only when they received doses equivalent to 200 and 300 kg N ha^-1^. The two cultivars showed a quadratic response (p ≤ 0.05) to TMR as N doses increased, resulting in the highest absolute values of 1.58 and 1.18 tillers 100 tillers^-1^ day^-1^ for Quênia and Tamani, respectively, corresponding to the highest N dose.

The variables LLS, LSR, NLL, TER, and TMR were affected (p ≤ 0.05) by the interaction of CV × SH (**Table 2**). The Tamani cultivar showed the highest LLS (17.63 days) in the full sun treatment (0% shading) compared with Quênia. With increasing shading levels, Tamani cultivar decreased linearly (p ≤ 0.05) on LLS, with means ranging from 17.63 to 14.50 days. This suggests that Tamani cultivars may be more vulnerable to the effects of increased shading. The LSR increased linearly (p ≤ 0.05) with increasing shading levels in the Tamani cultivar, whereas no significant difference (p > 0.05) was observed in the Quênia cultivar. The NLL was higher in Quênia than in Tamani when subjected to 0% shading. The NLL of Quênia decreased linearly (p ≤ 0.05) with increasing shading levels, whereas Tamani showed a quadratic effect (p ≤ 0.05) with the highest NLL at 30% shading.

**Table 2:** Effects of the interaction between cultivar and shading on morphogenetics and structural characteristics of tropical grasses of the genus *Megathyrsus*.

Cultivar	Shading (%)			Contrast	Mean	SEM
	0	30	50			
LLS (days)						
Quênia	11.24 b	13.71 a	12.09 a	ns	12.34	
Tamani	17.63 a	14.98 a	14.50 a	L	15.70	
Mean	14.44	14.35	13.29			
SEM						0.91
LSR (cm tiller^-1^ day^-1^)						
Quênia	0.091 a	0.110 b	0.115 b	ns	0.105	
Tamani	0.090 a	0.132 a	0.154 a	L	0.125	
Mean	0.090	0.122	0.134			
SEM						0.015
NLL						
Quênia	5.70 a	4.83 a	4.56 a	L	5.01	
Tamani	4.58 b	4.75 a	4.34 a	Q	4.56	
Mean	5.12	4.79	4.45			
SEM						0.09
TER (tillers 100 tyllers^-1^ day^-1^)						
Quênia	0.44 b	0.59 b	0.50 b	Q	0.51	
Tamani	0.50 a	0.88 a	0.61 a	Q	0.66	
Mean	0.47	0.74	0.55			
SEM						0.03
TMR (tillers 100 tyllers^-1^ day^-1^)						
Quênia	1.34 a	1.15 a	1.19 a	ns	1.23	
Tamani	1.08 b	1.11 a	1.11 a	ns	1.10	
Mean	1.21	1.13	1.15			
SEM						0.02

LLS, Leaf lifespan; LSR, leaf senescence rate; NLL, number of live leaves; TER, tiller emergency rate; TMR, tiller mortality rate; L, linear effect; Q, quadratic effect; ns, not significant (P>0.05); SEM, standard error of the mean. Means followed by the same letter within columns are not different (P>0.05).

The tiller emergence rate (TER) was higher in Tamani (p ≤ 0.05) at all shading levels when compared to Quênia (**Table 2**). Both cultivars showed a quadratic effect (p ≤ 0.05) on TER with increasing shading levels, with higher mean at intermediate levels of shade (30%). The Quênia cultivar showed higher TMR (1.34 tillers 100 tillers^-1^ day^-1^) under the no-shading treatment, whereas both cultivars had similar mean TMR at 30 and 50% shading levels. No significant effect (p>0.05) on TMR was observed for either cultivar with increasing shading levels.

The variables LSR, NLL, and TER were affected (p ≤ 0.05) by the NF × SH interaction (**Table 3**). Only the 0% shading treatment showed a linear increase in LSR as the dose of N increased, showing that the application of 30 and 50% nitrogen did not affect the LSR. When examining the effects of increased shading levels on the LSR of grasses, it was observed that a dose of 100 kg N ha^-1^ resulted in a quadratic (p ≤ 0.05) convex response, whereas doses of 200 and 300 kg N ha^-1^ provided an increasing linear effect.

**Table 3:** Effects of the interaction between nitrogen fertilization and shading on morphogenetics and structural characteristics of tropical grasses of the genus *Megathyrsus*.

Nitrogen fertilization	Shading (%)			Contrast	Mean	SEM
	0	30	50			
LSR (cm tiller^-1^ day^-1^)						
0	0.060	0.111	0.086	ns	0.860	
100	0.068	0.129	0.108	Q	0.102	
200	0.078	0.121	0.172	L	0.124	
300	0.108	0.126	0.173	L	0.136	
Contrast	L	ns	ns			
Mean	0.090	0.122	0.134			
SEM						0.20
NLL						
0	5.27	4.56	4.13	L	4.62	
100	5.08	4.91	4.75	ns	4.93	
200	4.99	4.94	4.59	ns	4.83	
300	5.14	4.77	4.39	L	4.77	
Contrast	ns	ns	Q			
Mean	5.12	4.79	4.45			
SEM						0.10
TER (tillers 100 tyllers^-1^ day^-1^)						
0	0.34	0.27	0.36	ns	0.32	
100	0.40	0.41	0.53	ns	0.45	
200	0.47	0.85	0.54	Q	0.62	
300	0.67	1.42	0.77	Q	0.95	
Contrast	L	L	L			
Mean	0.47	0.74	0.55			
SEM						0.06

LSR, leaf senescence rate; NLL, number of live leaves; TER, tiller emergency rate; L, linear effect; Q, quadratic effect; ns, not significant (P>0.05); SEM, standard error of the mean.

The quadratic effect of N dose on NLL was observed only under 50% shading (**Table 3**). The NLL decreased linearly (p ≤ 0.05) in the unfertilized treatment and in the treatment with 300 kg of N ha^-1^ with increasing shading levels. The TER showed an increasing linear effect at all levels of shading as N fertilization increased, demonstrating the importance of N input for grasses to continue issuing new tillers in unshaded and shaded plants. Only doses of 200 and 300 kg N ha^-1^ had a quadratic effect (p ≤ 0.05) on TER with increasing shading levels, with the highest mean values observed at 30% shading.

There were isolated effects of CV, NF, and SH (p ≤ 0.05) on the morphogenetic and structural variables LER, LAR, SER, and FLS (**Table 4**). The cultivar effect was observed for the variables LER and FLS, with the highest mean for these two variables, 6.21 cm leaf^-1^ day^-1^ and 25.36 cm, respectively, for the cultivar Quênia. N fertilization altered (p ≤ 0.05) LER, LAR, and SER variables, with a linear increase as N doses increased. Shading affected (p ≤ 0.05) LAR and SER with decreasing and increasing linear effects, respectively.

**Table 4:** Effects of cultivars, nitrogen fertilization, and shading on morphogenetic and structural characteristics, and on leaf anatomy variables of tropical grasses of the genus *Megathyrsus*.

Treatments	LER	LAR	SER	FLS	ADE	ABE	VBPS	VT	IS
Quênia	6.21 a	0.1447	0.53	25.36 a	0.60 a	0.87	100.48	17.48 b	0.07 a
Tamani	5.33 b	0.1536	0.50	23.24 b	0.52 b	0.78	100.01	18.92 a	0.05 b
SEM	0.19	0.0056	0.03	0.47	0.016	0.03	0.29	0.30	0.001
NF_0_	5.11	0.1271	0.38	24.09	0.59 a	0.86	102.14 a	21.57 a	0.06
NF_100_	5.96	0.1523	0.54	25.22	–	–	–	–	–
NF_200_	6.05	0.1567	0.52	24.17	–	–	–	–	–
NF_300_	5.96	0.1609	0.62	23.79	0.52 b	0.80	98.34 b	14.84 b	0.06
Contrast	L	L	L	ns	–	–	–	–	–
SEM	0.28	0.0077	0.04	0.72	0.016	0.04	0.29	0.30	0.001
SH_0_	5.92	0.1644	0.46	24.00	0.92 a	1.17 a	99.95	17.57 b	0.07 a
SH_30_	5.84	0.1436	0.52	24.80	–	–	–	–	–
SH_50_	5.55	0.1389	0.57	24.20	0.20 b	0.48 b	100.53	18.84 a	0.05 b
Contrast	ns	L	L	ns	–	–	–	–	–
SEM	0.25	0.0066	0.04	0.29	0.015	0.03	0.30	0.30	0.002

NF_0_, nitrogen fertilization 0 kg N ha^-1^; NF_100_, nitrogen fertilization 100 kg N ha^-1^; NF_200_, nitrogen fertilization 200 kg N ha^-1^; NF_300_, nitrogen fertilization 300 kg N ha^-1^; SH_0,_ 0% shading; SH_30,_ 30% shading; SH_50,_ 50% shading; LER, leaf elongation rate (cm tiller^-1^ day^-1^); LAR, leaf appearance rate (leaves tiller^-1^ day^-1^); SER, stem elongation rate (cm tiller^-1^ day^-1^); FLS, final leaf size (cm); ADE, adaxial epidermis (µm); ABE, abaxial epidermis (µm); VBPS, vascular bundle parenchyma sheath (µm^2^); VT, vascular tissues (µm^2^); IS, intercellular spaces (µm^2^); L, linear effect; ns, not significant (P>0.05); SEM, standard error of the men. Means followed by the same letter within columns are not different (P>0.05).

### 3.2 Leaf anatomy

**Figure 1:**
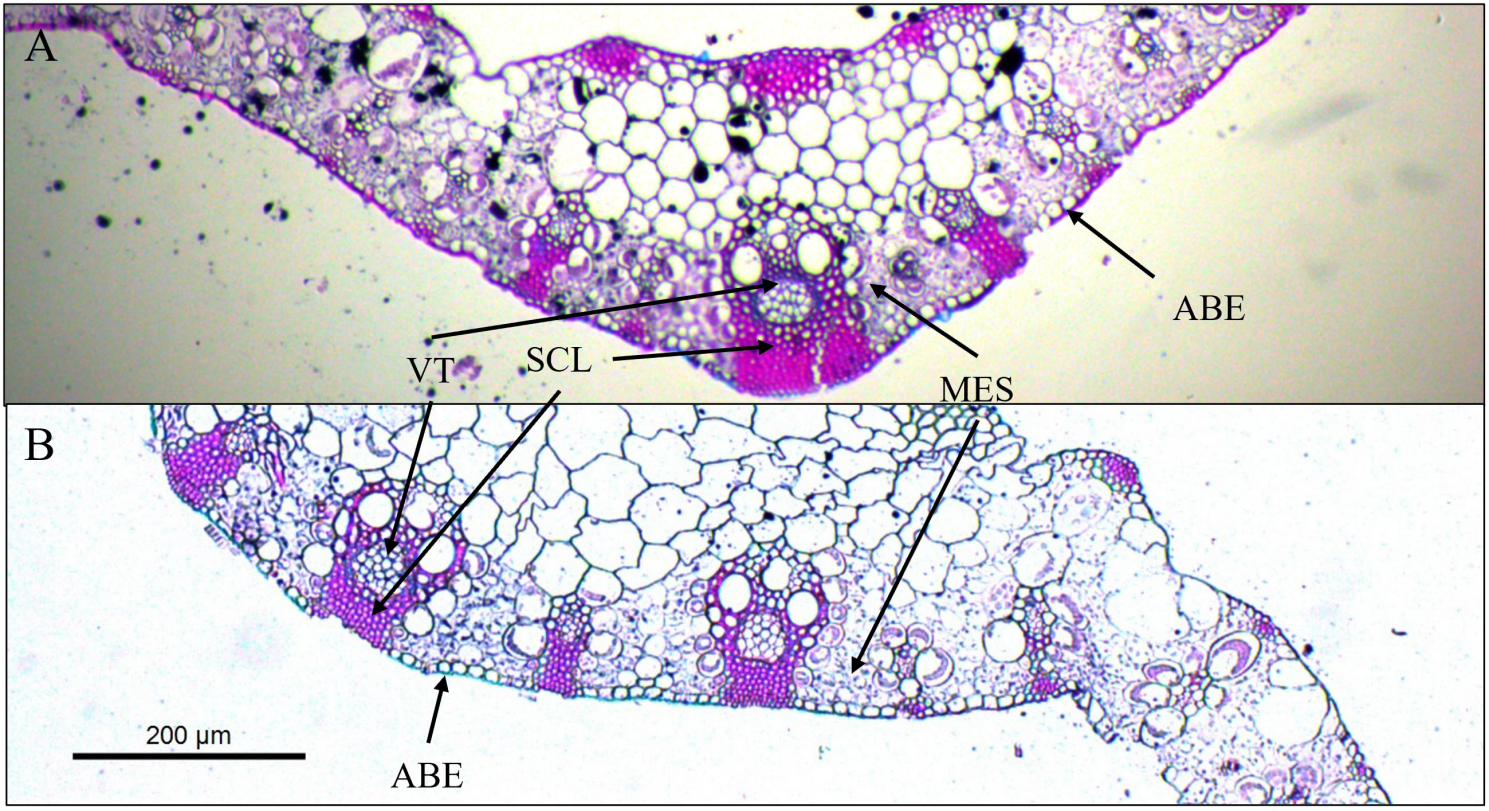
Cross section of leaf blade fragments of *Megathyrsus maximus* cv. Quênia, without fertilization **(A)** and with 300 kg of N ha^-1^ subject to 50% shading **(B)**. VT, vascular tissues; SCL, sclerenchyma; ABE, abaxial epidermis; MES, mesophyll.

The variables ADE, SCL, MES, CWPS, and TCSA were affected (p ≤ 0.05) by the CV × NF interaction (**Table 5**). ADE was higher in the Quênia cultivar (0.68 µm) compared to the Tamani (0.50 µm) when unfertilized. With the application of the 300 kg N ha^-1^, the Quênia cultivar had a reduction in ADE from 0.68 to 0.52 µm from the unfertilized treatment to the fertilized one. The Tamani cultivar showed higher SCL (8.53 µm^2^) compared to the Quênia (7.59 µm^2^) under 300 kg N ha^-1^. With fertilization, the Tamani cultivar showed an increase in SCL, from 7.67 µm^2^ without fertilization to 8.53 µm^2^ with 300 kg N ha^-1^. The mesophyll (MES) of both cultivars significantly increased (p ≤ 0.05) with fertilization (**Figure 1**). The Quênia cultivar demonstrated a higher mean CWPS (1.36 µm^2^) in unfertilized conditions, whereas the Tamani cultivar showed an increase in its CWPS from 0.96 to 1.30 µm^2^ with the application of 300 kg N ha^-1^. The Tamani cultivar fertilized with 300 kg N ha^-1^ showed higher TCSA (199.22 µm^2^) compared to BRS Quênia (194.44 µm^2^). From the treatment without fertilization to the treatment with 300 kg N ha^-1^, only the TCSA of the Quênia cultivar decreased from 201.00 to 194.44 µm^2^.

**Table 5:** Effects of cultivar and nitrogen fertilization interaction on leaf anatomy variables of tropical grasses of the genus *Megathyrsus*.

Cultivar	Nitrogen fertilization (kg ha^-1^)		Mean	SEM
	0	300		
ADE (µm)				
Quênia	0.68 aA	0.52 bA	0.60	
Tamani	0.50 aB	0.53 aA	0.52	
Mean	0.59	0.52		
SEM				0.23
SCL (µm^2^)				
Quênia	8.10 aA	7.59 aB	7.85	
Tamani	7.67 bA	8.53 aA	8.10	
Mean	7.88	8.06		
SEM				0.20
MES (µm^2^)				
Quênia	66.68 bA	71.53 aA	69.11	
Tamani	65.95 bA	73.00 aA	69.47	
Mean	66.32	72.27		
SEM				0.50
CWPS (µm^2^)				
Quênia	1.36 aA	1.28 aA	1.32	
Tamani	0.96 bB	1.30 aA	1.13	
Mean	1.16	1.29		
SEM				0.03
TCSA (µm^2^)				
Quênia	201.00 aA	194.44 bB	197.72	
Tamani	200.25 aA	199.22 aA	199.73	
Mean	200.62	196.83		
SEM				0.837

ADE, adaxial epidermis; SCL, sclerenchyma; MES, mesophyll; CWPS, cell wall parenchyma sheath; TCSA, total cross-sectional area; SEM, standard error of the mean; Means followed by the same uppercase letter within columns and the same lowercase letter within lines are not different (P>0.05).

The variables CWPS and TCSA were affected (p ≤ 0.05) by the CV × SH interaction, and the variables VT, MES, and IS were affected by the NF × SH interaction (**Table 6**). The Quênia cultivar showed the highest CWPS when not shaded (1.66 µm^2^) compared to Tamani (1.34 µm^2^). The two cultivars showed reduced CWPS when subjected to 50% shade. This reduction could potentially enhance the digestibility of the leaf blades when consumed by ruminants. TCSA was higher (201.58 µm^2^) in the Tamani cultivar compared to BRS Kenya (197.52 µm^2^) under unshaded conditions. However, only Tamani showed a reduction in TCSA from 201.58 to 197.88 µm^2^ when subjected to 50% shading.

**Table 6:** Effects of interaction between cultivar and shading, and nitrogen fertilization and shading on leaf anatomy variables of tropical grasses of the genus *Megathyrsus*.

	Shading (%)		Mean	SEM
	0	50		
Cultivar	CWPS (µm^2^)			
Quênia	1.66 aA	0.98 bA	1.32	
Tamani	1.34 aB	0.92 bA	1.13	
Mean	1.50	0.95		
SEM				0.04
	TCSA (µm^2^)			
Quênia	197.52 aB	197.92 aA	197.72	
Tamani	201.58 aA	197.88 bA	199.73	
Mean	199.55	197.90		
SEM				0.738
Nitrogen fertilization (kg ha^-1^)	VT (µm^2^)			
0	20.00 bA	23.14 aA	21.57	
300	15.14 bB	14.54 bA	14.84	
Mean	17.57	18.84		
SEM				0.71
	MES (µm^2^)			
0	67.43 bA	65.20 bB	66.32	
300	72.10 aA	72.43 aA	72.27	
Mean	69.77	68.82		
SEM				0.71
	IS (µm^2^)			
0	0.066 aA	0.055 bB	0.061	
300	0.063 aA	0.061 aA	0.062	
Mean	0.065	0.058		
SEM				0.002

CWPS, cell wall parenchyma sheath; TCSA, total cross-sectional area; VT, vascular tissues; MES, mesophyll; IS, intercellular spaces; SEM, standard error of the mean; Means followed by the same uppercase letter within columns and the same lowercase letter within lines are not different (P>0.05).

It was observed that VT of the leaf blade was greater (23.14 µm^2^) when the grasses were shaded at 50% and unfertilized (**Table 6**). When fertilized, the grasses showed a decrease in VT under both full sun and 50% shade. The MES, only in the treatment without fertilization, reduced from 67.43 to 65.20 µm^2^ with increased shading from 0 to 50%, which was also observed for the IS, which decreased from 0.066 to 0.055 µm^2^. **Table 4** shows the means of leaf anatomy variables affected by the isolated factors of cultivar, nitrogen fertilization, or shading (p ≤ 0.05). The Quênia cultivar exhibited a higher ADE thickness (0.60 µm^2^), a smaller VT (17.48 µm^2^), and larger IS (0.07 µm^2^) (p ≤ 0.05) compared to Quênia. N fertilization decreased ADE, VBPS, and VT, resulting in a 31.20% decrease in VT.

There was a reduction (p ≤ 0.05) in ADE, ABE, and IS as shading levels increasing from 0 to 50% (**Table 4**), of 78% for ADE and 58% for ABE. This establishes that shade has a notable effect on the epidermal thickness of leaf blades in grasses. On the other hand, VT increased from 17.57 to 18.84 µm^2^ when the plants were shaded.

## 4 Discussion

### 4.1 Morphogenetics and structural characteristics

When unfertilized or given the lowest dose of fertilizer (100 kg N ha^-1^), the Quênia cultivar proved to be more efficient than the Tamani cultivar in terms of the time elapsed between the appearance of two consecutive leaves on the tiller (phyllochron) (**Table 1**). As the N dose increased, the phylochronology of the Quênia cultivar remained unchanged, whereas that of the Tamani cultivar decreased linearly. Therefore, Quênia was less affected by nitrogen in terms of reducing phyllochronos, which could be attributed to the genetic characteristics of this cultivar. Martuscello et al. (2018) and Martuscello et al. (2019), who also evaluated Quênia and Tamani cultivars, found that N impacts plant growth, enhancing its capacity to regrow after cutting. This is due to the rapid restoration of its photosynthetic apparatus, which determines its survival in the plant community.

In relation to the TER, the Tamani cultivar showed higher rates than Quênia (**Table 1**) when fertilized. This could potentially be attributed to the inherent characteristics of the cultivar, such as having smaller and more tillers, particularly when N-fertilized. The increase in TER of the two cultivars may be linked to the ability of N to stimulate tillering. N tends to increase the ability of fertilized plants to form axillary buds and initiate the corresponding tillers (Bergoli et al., 2019). Thus, applying N to pastures with tropical grasses can increase the growth of axillary buds, resulting in a higher number of new tillers and increased rate of tiller emergence.

Similar to TER, TMR increased with increasing N doses in both cultivars (**Table 1**) as N induced not only tiller emergence but also mortality by increasing tissue flow. However, Quênia showed higher mortality and lower tiller emergence at doses of 200 and 300 kg N ha^-1^, indicating that it would be appropriate to use the Tamani cultivar at higher N doses. Similar results were observed by Bergoli et al. (2019) and Lopes et al. (2016) in other tropical grasses belonging to the genera *Urochloa* and *Megathyrsus*.

The leaf lifespan (LLS) of Quênia was 36% lower than that of Tamani when exposed to full sun (**Table 2**), whereas the LLS of the two cultivars were the same when shaded at 30 and 50%. The Tamani cultivar exhibited a linear decrease in LLS as the shade increased, demonstrating its sensitivity to reduced light exposure. This was reinforced by Tamani’s senescence (LSR), which increased linearly with increasing shade levels. These results indicate that, under more intense shade, the Tamani cultivar likely had trouble maintaining its metabolism and photosynthetic processes. LSR, which is characterized by a reduction in chlorophyll levels in plants, can be accelerated by stresses such as high temperatures, reduced radiation, excess water, and water and mineral deficits (Calbo et al., 1989). Townsend et al. (2013) evaluated the morphogenetic and structural characteristics of Brachiaria subjected to different shading levels (0, 20, and 50%) and observed a linear increase in leaf senescence with increasing shading levels, with values ranging from 0.043 to 0.086 cm leaf^-1^ tiller^-1^.

The reduction in NLL in the two cultivars subjected to 50% shading (**Table 2**) could be explained by the increase in leaf senescence observed with a reduction in luminosity. The comparison of NLL between cultivars was closely related to the genetics of each plant. This is a genetically determined variable, but it responds to variations in climate and soil and can decrease under stressful conditions. Plants when in shady conditions perform adaptive phenotypic plasticity, reducing the number of leaves or changing tillering patterns (Abraham et al., 2014). In contrast, the higher TER in Tamani than in Quênia at all shading levels (**Table 2**) may have been a response of Tamani to compensate for the effects of shading. For both cultivars, TER increased up to 30% shading and then decreased at 50% shading. Under more severe shading conditions, the grasses tended to reduce the emergence of new leaves; consequently, there was a reduction in the emergence of tillers, as these two variables were correlated. When a new leaf emerges, it is possible that the axillary or lateral bud gives rise to a new tiller. Quênia is probably less tolerant to environmental conditions without shade, such as high temperatures, which may have led to an increase in TMR and a reduction in LLS (**Table 2**).

An increase in LSR with an increase in N was observed only when there was no shading (**Table 3**), suggesting that shaded grasses could regulate leaf senescence with the application of fertilizer, which is an interesting strategy (Silva et al., 2019). This is likely due to the higher soil humidity in shaded areas compared to that in unshaded soils, which can favor the availability of N in the soil (Wilson, 1996). The increase in LSR with increased fertilization in the non-shaded environment may also be related to the observation that plants exhibit low levels of leaf senescence when deprived of N or with minimal input. This may be a survival tactic, possibly caused by reduced metabolism, as N has been shown to hasten tissue flow in tropical forage plants (Braz et al., 2011; Freitas et al., 2012).

The linear increases in LSR with higher fertilization (200 and 300 kg N ha^-1^) when the grasses were shaded (**Table 3**) were probably due to the effect of N overshading, as these are considered high doses. N fertilization reduces the availability of photosynthetically active radiation in the lower part of the plant by accelerating the flow of grass tissues through higher LAR and LER (**Table 4**), thus providing favorable conditions for the leaf senescence process as a direct result of the established level of shading (Gastal et al., 2010). In the absence of fertilization, the plants kept their leaves alive longer (LLS) (**Table 4**) to the detriment of the expansion of new leaves; that is, the senescence process was accelerated with increasing doses of N, reducing LLS as a result of greater tissue renewal in the plants.

The effect of shading on NLL in tillers was inversely related to LSR, which tended to increase with increasing grass shading, mainly because of the reduction in solar radiation reaching the leaves. Thus, NLL in tillers decreased in shaded pastures, which must be considered when using this variable as a practical index to guide pasture management in shaded environments, such as silvopastoral system.

The observation of a quadratic effect at doses of 200 and 300 kg N ha^-1^ and no effect at doses of 0 and 100 kg N ha^-1^ with increasing shade (**Table 3**) in the variable TER indicates that higher N inputs promoted the emergence of tillers up to 30% shade, probably by activating lateral or axillary buds. At 50% shade, the emergence of tillers was significantly reduced, as there was a reduction in LAR, and these two variables were positively and directly correlated. In addition, at high levels of shade (50%), the lower intensity of radiation and reduced ratio of red to far-red wavelengths inhibit tillering (Deregibus et al., 1983). Davies et al. (1983) showed that in shaded plants, a greater amount of photoassimilates is allocated to the growth of existing tillers than to the development of new tillers.

Both cultivars have erect or cespitose growth, however, the leaves of the Quênia cultivar were naturally larger and wider than those of the Tamani cultivar. This characteristic explains the higher LER (6.21 cm tiller^-1^ day^-1^) and FLS (25.36 cm) observed in the Quênia cultivar (**Table 4**). Similarly, Paciullo et al. (2016) observed higher values of LER (3.6 cm tiller^-1^ day^-1^) and FLS (34.3 cm) in the Tanzania cultivar than in the Massai cultivar (LER=2.49 cm tiller^-1^ day^-1^ and FLS=23.6 cm), both of which also belong to the genus *Megathyrsus* and have contrasting morphological characteristics.

The increase in LER, LAR, and SER variables with increasing N fertilization (**Table 4**) has also been observed by several authors (Paciullo et al., 2016; Martuscello et al., 2019; Oliveira et al., 2020; Cunha et al., 2022) and is mainly due to the high demand for this nutrient in meristematic zones and the acceleration of tissue flow in grasses, in addition to the participation of N in the synthesis of new tissues (Gastal and Nelson, 1994). Nitrogen also increasing the plant’s gas exchange, favoring its development, since the higher rate of transpiration accelerates the enzymatic activity of nitrogen utilization and the absorption of water by the plant’s roots, thus accelerating the elongation of plant components plant (Lopes et al., 2016). In Tanzania and Massai cultivars, Paciullo et al. (2016) observed increasing values of LER and SER, with means from 2.4 to 3.5 cm tiller^-1^ day^-1^ and from 0.095 to 0.157 cm tiller^-1^ day^-1^, respectively, in unfertilized treatments up to treatments with a dose of 150 kg N ha^-1^. Stem elongation in pastures treated with high N doses may be related to the self-shading effect of the upper leaves on the lower leaves of the tillers, which promotes a greater need for the elongation of both leaves and stems. Therefore, under these conditions, plants may prioritize stem elongation and place leaves in higher layers to capture more light.

The decrease in LAR of the cultivars with increasing shading levels (**Table 4**) may be related to the increase in SER with increasing shading as, under shaded conditions, tropical grasses use the mechanism of stem and leaf elongation to achieve greater luminosity, and the time between the initiation of the leaf primordium in the apical meristem and the subsequent appearance of the leaf above the stem represents a growth period that can be influenced by the length of the pseudocolm (Skinner and Nelson, 1995). Moreover, the greater the shading imposed on the grass, the more physiological changes occur, such as a decrease in stomatal conductance and photosynthetic rate, causing the plant to reduce its energy production to issue new leaves (Cruz et al., 2024). These results corroborate those of Paciullo et al. (2016), who evaluated Tanzanian and Massai grasses under different shading levels (0%, 37%, and 58%) and also found a lower leaf emergence rate and greater stem elongation under shaded conditions.

### 4.2 Leaf anatomy

The anatomical characteristics of forage grasses are highly dependent on the environment in which they are grown and the phenotypic ability of the grass to adapt to different environments. These anatomical changes can vary not only between genera, but also between cultivars of the same species (Gobbi et al., 2011; Taiz and Zeiger, 2013).

Differences in the proportion of epidermis in the leaf blades between cultivars can be attributed to the inherent genetic characteristics of each cultivar. Thus, the Quênia cultivar showed a higher mean only in the ADE (0.60 µm) compared to Tamani (0.52 µm) (**Table 4**), while the thickness of the ABE was the same in both cultivars. The effect of nitrogen fertilization on ADE was only observed in Quênia, which decreased from 0.68 to 0.52 µm with fertilization of 300 kg N ha^-1^. The highest LER observed at the dose of 300 kg ha^-1^ explains the result, since the expansion of the cells decreased the thickness of the epidermal tissues, as observed in the study (Koch et al., 2019). Moreover, higher doses of N can probably reduce the lignification of the epidermis, providing more digestible leaf tissues as the expansion of cells can reduce the thickness of the epidermis. According to Mauseth (1988), ADE changes the most when N doses are applied owing to the occurrence of buliform cells.

The main characteristic of sclerenchyma is the presence of thick secondary walls, whether lignified or not, with homogeneous and regular thickening of the cell walls. It is a supporting tissue present in the periphery or innermost layers of the organ (Leroux et al., 2018). The sclerenchyma cells of grasses develop a thick secondary wall that becomes lignified with increasing plant age, besides needing to strengthen its cell wall, due to the increase in productivity caused by nitrogen fertilization (Lopes et al., 2020). Comparing the cultivars, the area occupied by the sclerenchyma cells was greater only in the Tamani cultivar when it was fertilized with 300 kg N ha^-1^ (8.53 µm^2^) (**Table 5**). This difference could be related to the genetic characteristics of each cultivar and the fact that Tamani increased SCL when fertilized.

The response of sclerenchyma to nitrogen fertilization varied significantly. Basso et al. (2014), studying *Megathyrsus maximus* cv. Milênio observed a decrease in sclerenchyma from 2.5 to 1.5% in leaf blades as the N dose increased from 0 to 400 kg ha^-1^. Similarly, Lajús et al. (2014) investigated Axonopus jesuiticus under N doses of 0, 100, 200, 300, and 400 kg ha^-1^ and observed a decrease in the sclerenchyma percentage from 25 to 15%. This variation in the sclerenchyma due to nitrogen fertilization can be attributed to the intensity of nitrogen interference in regions occupied by other tissues, such as photosynthetic tissues.

Mesophyll consists of parenchymatous tissues in plant leaves located between the upper and lower epidermis. The majority of this tissue in all leaves is composed of chlorenchymatous cells, although it degrades more easily in grass than in other plant tissues. The increase in the MES of the two cultivars with the use of N fertilization (**Table 5**) may be associated with the large number of chloroplasts found in the mesophyll cells, in these chloroplasts are located which chlorophyll pigments are responsible for transforming the light energy required for the synthesis of metabolites such as sucrose and starch (Taiz and Zeiger, 2013; Evans, 2020). Thus, the increase in mesophyll area owing to N fertilization, coupled with an increase in chlorophyll content, is linked to enhanced photosynthetic capacity at the leaf level. Therefore, due to the greater photosynthetic rate of the plant the plants present a more accelerated development, which explains why fertilized plants have a greater LER and LAR. When under shading stress, mesophyll density tends to decrease, so that the plant can capture more light, thus reducing the emergence of new leaves and maintaining the leaves that are present for longer, leaving the leaves flatter to increase their area. foliar (La Riva et al., 2016).

Vascular bundles in plants are separated by the mesophyll, which contains sparsely arranged cells. C4 grasses, however, feature a parenchymatous sheath surrounding the vascular bundles, consisting of large cells with walls up to five times thicker than the mesophyll cells. These sheath cells are rich in chloroplasts and participate in the photosynthetic processes. Their characteristics vary from species to species and are affected by environmental and management conditions. The area occupied by the cell wall parenchyma sheath (CWPS) was lower in the Tamani cultivar without N fertilization (0.96 µm^2^) compared to Quênia (1.36 µm^2^) (**Table 5**). When 300 kg N ha^-1^ was applied, the Tamani cultivar showed a 26% increase in the CWPS area, which may be attributed to an increase in the chloroplast concentration in these cells. The increase in N may affect the CWPS, potentially leading to a higher lignin content in these cells to provide additional support to the plant.

The shading effect in reducing CWPS in both cultivars (**Table 6**) may be due to the higher incident temperature on the grasses under full sun than when shaded at 50%. Temperature is an environmental factor that has the greatest influence on the thickening of CWPS, hindering the digestibility of the parenchymatous sheath of bundles when consumed by ruminant animals. The Quênia cultivar exhibited greater sensitivity to this temperature than the Tamani cultivar, suggesting that it may have lower nutritional value at high temperatures.

The use of N generally resulted in a decrease in the total cross-sectional area (TCSA) occupied by leaf tissues, which was mainly attributed to a decrease in the TCSA in the Quênia cultivar (**Table 5**). These variations may arise from the location and management of the plant, as the plant employs its phenotypic plasticity to augment or diminish structures and cells for the optimal utilization of resources, specifically photosynthesis (Gobbi et al., 2011).

The decrease from 21.57 to 14.84 µm^2^ of vascular tissue (VT) when the grasses were fertilized (**Table 4**) may have been due to an increase in the leaf mesophyll, given that the mesophyll cells are more compactly irradiated around the VBPS and vascular bundles. The mesophyll is rich in N, and as previously reported, tends to increase its occupancy in the leaf blade due to the higher concentration of chloroplasts in its cells. Thus, it can be inferred that by reducing the VT and increasing the MES through use of N fertilization, the nutritional value of the studied cultivars may be improved. This improvement in nutritional value may reflect greater digestibility when consumed by ruminants. The larger area covered by the VT in the Tamani cultivar (18.92 µm^2^) may have contributed to the lower IS (0.05 µm^2^) compared to Quênia (**Table 4**). These variations in tissue area between cultivars may be related to the morphology of their leaf blades. While the leaf blades of Quênia were longer and wider, those of Tamani were smaller and narrower. This difference in VT may be due to the fact that longer and wider leaf blades have a more pronounced central vein and more secondary veins, where the VT are located. In relation to VBPS, it is the fact that older leaves have higher lignin levels (Khaled et al., 2006), which explains the fact that the treatment without fertilization presents higher VBPS, since the LLS of these plants is higher.

The decrease in ADE and ABE in shaded grasses may be attributed to the reduced luminosity reaching the leaves. Consequently, grasses tend to exhibit an increase in specific leaf area (leaf area/leaf mass), which correlates with the anatomical changes that can occur in shaded plants, including a reduction in the thickness of the cuticles and epidermis (Deinum et al., 1996).

## 5 Conclusion

This study supports the hypothesis that the interaction between N fertilization and shading affects the growth patterns and leaf anatomy of *Megathyrsus* cultivars.

The Tamani cultivar demonstrated greater tolerance and efficiency with regard to canopy structure under 30% shading, whereas Quênia was less tolerant and efficient under both 30 and 50% shading when morphogenic and structural variables were analyzed.

N fertilization promotes alterations in leaf anatomy, resulting in a decrease in tissues that are less digestible to ruminants, such as the epidermis and vascular tissues, and an increase in more digestible tissues, such as the mesophyll. Shading decreased the epidermis but amplified the space occupied by vascular tissues in the studied cultivars. The Tamani cultivar altered the anatomy of its leaves in response to shading, whereas the Quênia cultivar modified its leaf anatomy in response to N fertilization. This reinforces the suggestion that Tamani is more suitable for use in silvopastoral systems. However, it is important to note that this study was conducted under artificial and controlled shading conditions without the presence of any trees competing for moisture and nutrients. Therefore, recommendations based on these findings should be approached with caution when applied in real-world field conditions.

## Data availability statement

The original contributions presented in the study are included in the article/supplementary material. Further inquiries can be directed to the corresponding author.

## Author contributions

AL: Data curation, Formal Analysis, Investigation, Methodology, Project administration, Visualization, Writing – original draft, Writing – review & editing. NL: Data curation, Formal Analysis, Investigation, Methodology, Writing – review & editing. AR: Supervision, Writing – original draft. FD: Supervision, Writing – original draft. TS: Supervision, Writing – original draft. CF: Supervision, Writing – original draft. NS: Data curation, Investigation, Project administration, Supervision, Writing – original draft. WS: Conceptualization, Data curation, Formal Analysis, Methodology, Project administration, Supervision, Visualization, Writing – original draft, Writing – review & editing.
